# *Mycoplasma hyorhinis* and *Mycoplasma hyosynoviae* dual detection patterns in dams and piglets

**DOI:** 10.1371/journal.pone.0209975

**Published:** 2019-01-03

**Authors:** Luiza R. Roos, Meera Surendran Nair, Aaron K. Rendahl, Maria Pieters

**Affiliations:** Department of Veterinary Population Medicine, College of Veterinary Medicine, University of Minnesota, St. Paul, Minnesota, United States of America; University of Connecticut, UNITED STATES

## Abstract

*Mycoplasma hyorhinis* and *M*. *hyosynoviae* are agents associated with arthritis in pigs. This study investigated the tonsillar detection patterns of *M*. *hyorhinis* and *M*. *hyosynoviae* in a swine population with a history of lameness. The plausibility of dual PCR detection of these agents in dams at one and three weeks post-farrowing and their offspring at the same time was determined. The association between *M*. *hyorhinis* and *M*. *hyosynoviae* detection in piglets and potential development of lameness in wean-to-finish stages was evaluated by correlating individual piglet lameness scores and PCR detection in tonsils. Approximately 40% of dams were detected positive for *M*. *hyorhinis* and *M*. *hyosynoviae* at both one and three weeks post-farrowing. In first parity dams, *M*. *hyorhinis* was detected in higher proportions (57.1% and 73.7%) at both weeks of sampling compared to multi-parity dams. A lower proportion of first parity dams (37.5%) were detected positive at week one with *M*. *hyosynoviae* and an increase in this proportion to 50% was identified in week three. Only 8.3% of piglets were detected positive for *M*. *hyorhinis* in week one compared to week three (50%; *p*<0.05). The detection of *M*. *hyosynoviae* was minimal in piglets at both weeks of sampling (0% and 0.9%). Lameness was scored in pigs 5–22 weeks of age, with the highest score observed at week 5. The correlation between PCR detection and lameness scores revealed that the relative risk of developing lameness post-weaning was significantly associated with detection of *M*. *hyorhinis* in piglets at three weeks of age (r = 0.44; *p*<0.05).The detection pattern of *M*. *hyorhinis* and *M*. *hyosynoviae* in dams did not reflect the detection pattern in piglets. Results of this study suggest that positive detection of *M*. *hyorhinis* in piglets pre-weaning could act as a predictor for lameness development at later production stages.

## Introduction

*Mycoplasma hyorhinis* and *M*. *hyosynoviae* are considered commensal microorganisms of the upper respiratory tract and tonsils of pigs [1,2]. These bacterial agents are “facultative” pathogens of swine known to cause arthritis and/or polyserositis [3]. Often, *M*. *hyorhinis* associated arthritis and polyserositis are reported in nursery pigs, especially between six and ten weeks of age, [4,5] and infection in pigs older than three months of age usually results in mild arthritis [6]. *Mycoplasma hyosynoviae* is mostly identified as a causative agent of arthritis [2] in finishing pigs [7,8] and the lesions are known to be restricted to the joints together with the involved synovial membranes. Therefore, infectious arthritis, particularly *Mycoplasma*-associated clinical arthritis is a concern to swine producers, and can impact both animal well-being, and performance during all stages of swine production.

It has been suggested that *M*. *hyorhinis* can persist chronically in joints [9,10], however, bacterial isolation of the microorganism from clinically affected pigs might not be definitive for diagnosis of disease [11]. Similarly, *M*. *hyosynoviae* might also be detected in joints with no apparent clinical presentation or macroscopic joint lesions observed [8,12]. The prevalence of *M*. *hyorhinis* and *M*. *hyosynoviae* prior to weaning has been described using several sample types and tests, such as bacterial culture from tonsil tissue and synovial fluids [2,13,14], serum tested by ELISA and complement fixation [12,13,15], as well as oral fluids and nasal swabs tested by real-time PCR [3,16]. Furthermore, *M*. *hyorhinis* has also been reported to be detected from pneumonic lungs in fattening pigs by real time PCR[17]. Nevertheless, the mechanisms of systemic spread and disease development of these two microorganisms are still unknown. Additionally, other microorganisms including, but not limited to, *Haemophilus parasuis*, *Erysipelothrix rhusiopathiae*, and *Streptococcus suis* can produce a similar type of arthritis in pigs [18].

*Mycoplasma hyorhinis* is assumed to be transmitted from dams to piglets shortly after birth [19], with decreased transmission between piglets after 8 weeks of age [20]. In the case of *M*. *hyosynoviae*, the likelihood of transmission is mostly limited to pigs older than 4–8 weeks of age [13], with various investigations showing detection of *M*. *hyosynoviae* in the tonsils of suckling pigs [11,14,21,22]. However, limited studies have investigated whether these two microorganisms are associated at any point in colonizing newborn pigs, or whether one bacterial species limits colonization with the other. Therefore, it was hypothesized that *M*. *hyorhinis* and *M*. *hyosynoviae* can be detected simultaneously in dams and their piglets using PCR from tonsillar swabs prior to weaning. Thus, the objectives of this study were to determine the dual detection patterns of *M*. *hyorhinis* and *M*. *hyosynoviae* in dams and piglets prior to weaning, and to evaluate the association between the detection of the two bacterial species and their correlation with pig lameness in later production stages. To the best of our knowledge, this is one of the first studies looking at the detection pattern of both microorganisms in tonsils of dams and piglets in the pre-weaning phase.

## Materials and methods

### 1. Ethics statement

Conventional crossbred lines of sows and piglets were included in this study. Animals were sampled following protocols approved by the University of Minnesota Institutional Animal Care and Use Committee (IACUC approval number: *1404-31453A)* and handled according to the sow farm standard procedures.

### 2. Animals and housing

A 2,000-sow farm located in the Midwest, USA, with unknown *M*. *hyosynoviae* prevalence at enrollment, but with a history of clinical disease (based on attending Veterinarian’s clinical evaluation and PCR positive diagnosis results) in the production system was selected for this investigation. All animals in this study were housed under commercial conditions and management practices standard in the swine industry. Gilts were purchased from an external multiplier. During the study period, the sow farm was negative for *M*. *hyopneumoniae* and endemically infected for porcine reproductive and respiratory syndrome virus.

According to sow-farm management practices, dams were vaccinated against porcine parvovirus, *E*. *rhusiopathiae*, and *Leptospira spp*. three weeks prior to farrowing. In general, piglets were treated with a combination of lincomycin, dexamethasone, and flunixin as a broad spectrum prophylactic measure to decrease lameness signs caused by common arthritogenic pathogens like *S*. *suis*. Nevertheless, it is important to mention that the dams and piglets enrolled in this study did not receive antimicrobial treatments, one month prior to, and throughout the course of the study. However, all piglets were administered farm immunizations. Piglets were processed (castrated and dock-tailed) within three days of age, cross-fostered if needed, and vaccinated at processing and one day prior to weaning against porcine circovirus type 2. The duration of the lactation period was between 18–23 days of age, with an average of 21 days.

For this study, one hundred- twenty piglets, individually ear-tagged, with even distribution by gender, were randomly selected at three days of age from 30 dams of various parities. Dams were randomly selected after stratification by parity. The sample size was estimated based on a 10% expected prevalence and a 95% confidence. The estimates for sensitivity and specificity of PCR in oral fluids were derived from previous studies conducted [3, 16]. However, one of the dams was culled due to agalactia, leading to a final dam sample size of 29. At the end of the study, all animals were retained in the farm and completed their production cycle. A client consent form was approved and signed by the swine producer prior to the start of the investigation.

### 3. Experimental design

**Fig 1:**
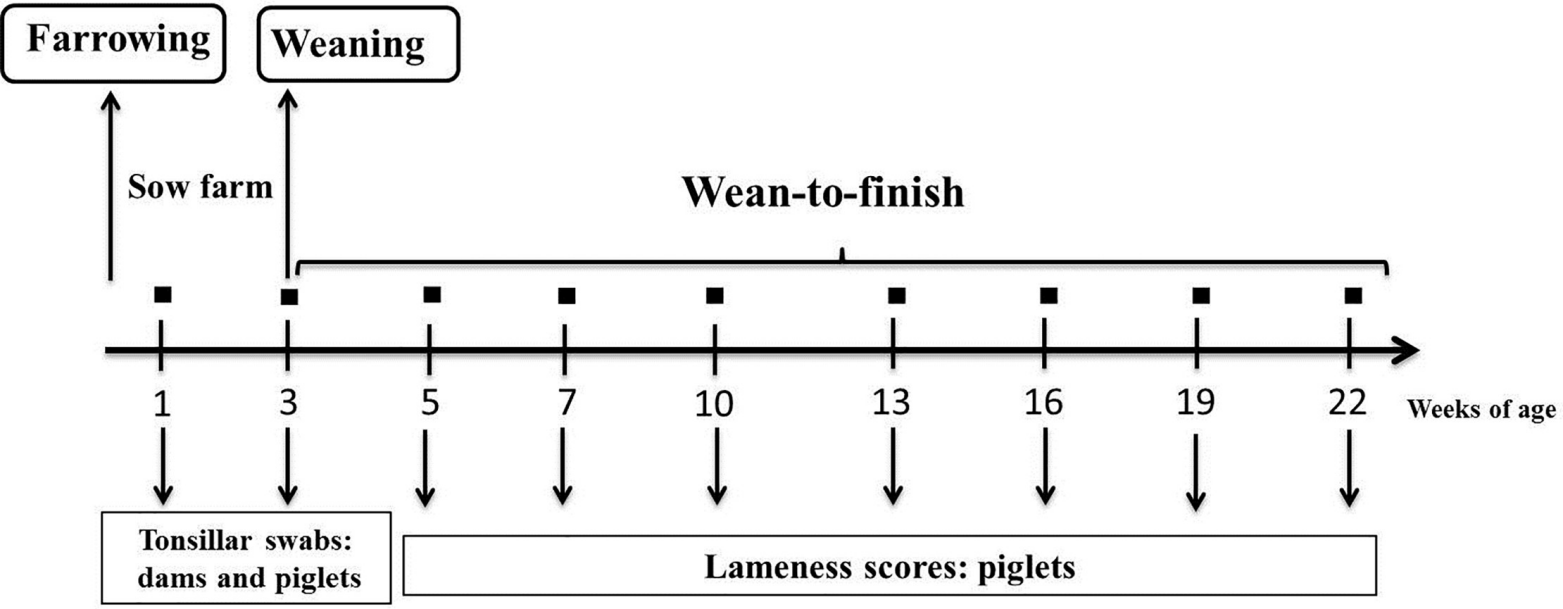
Study experimental design. Dams (n = 29) and piglets (n = 120) were sampled at one and three weeks post-farrowing using tonsillar swabs. Piglets (n = 60) were followed wean-to-finish and lameness was individually scored at 5, 7, 10, 13, 16, 19 and 22 weeks of age.

A graphical representation of the experimental design is shown in Fig 1. Tonsillar swabs were collected in the farrowing rooms from dams and piglets at one and three weeks post-farrowing. The overall goal of this study was to detect the pathogens in dams and piglets just after farrowing and just before weaning. Therefore, sampling times were chosen in order to avoid disruption of routine farm management practices. Sampling on week one post-farrowing occurred after processing and cross-fostering of piglets, whereas sampling on week three occurred prior to weaning. Further, selected weaned piglets (n = 60) were observed throughout 22 weeks of age and the lameness scores were recorded at weeks 5, 7, 10, 13, 16, 19 and 22. Approximately two piglets per dam were followed for lameness scoring.

### 4. Sample collection

The tonsillar swab collection process involved the use of a mouth gag and a headlamp to aid in the location of the tonsils in the mouth cavity. Tonsillar swabs were collected by rubbing a sterile swab (BBL CultureSwab, Sparks, MD, USA) on the tonsils surface. The process was performed in a similar way regardless of the age, and pigs were manually restrained during collection.

After weaning and at the wean-to-finish barn, lameness scores were determined individually by evaluating the selected pigs. Lameness score evaluation was performed by the same investigator throughout the study, using a 0 to 4 scale as previously proposed [8]. Briefly, a score of 0 described a scenario where the pig gets up immediately from a lying position and moves freely in the pen with balanced weight on all four limbs. For score 1, pig rises immediately but a reluctant movement is observed, with short steps and uneven distribution of body weight. For score 2, pig moves slowly in the pen with short steps and reduced weight in the sore limb, or pig rises slowly and the affected limb was not weight bearing. Score 3, the pig is reluctant to rise, with muscle shivering when standing and lifts the sore limb from the floor, or pig refuses to walk or walks on three limbs only. Finally, for score 4, pig only rises when forced and when standing has marked signs of pain (e.g. reluctance to move, limping and vocalization).

### 5. Sample processing and testing

Tonsillar swabs were transported to the laboratory and stored at -20°C until use. DNA was extracted using MagMAX-96 Viral RNA isolation kit and MagMAX Express-96 Magnetic Particle Processor (Life Technologies, Grand Island, NY, USA). For *M*. *hyorhinis* genetic material detection, a real-time PCR was performed with QuantiFast Probe PCR kit (Qiagen Inc., Germantown, MD, USA), according to manufacturer’s protocol, employing custom primers and probe [23]. Similarly, a real-time PCR assay was performed to detect *M*. *hyosynoviae* with Path-ID qPCR Master Mix Kit (Life Technologies, Grand Island, NY, USA), according to manufacturer’s protocol. The primers and probes were synthetized based on the Standard Operating Procedures (SOP) routinely followed at the University of Minnesota, Veterinary Diagnostic Laboratory for *M*. *hyosynoviae* detection (UMN-VDL SOP .0078). Samples were considered positive by real-time PCR for *M*. *hyorhinis* and *M*. *hyosynoviae* when Ct ≤ 37.

### 6. Statistical analysis

The proportion of dams and piglets detected with *M*. *hyorhinis* and *M*. *hyosynoviae* genetic material in tonsillar swabs were compared separately within the week of sampling using a two-sample test for equality of proportions with 95% confidence interval. For the proportions with zero detection, the Clopper-Pearson method was used to determine the confidence interval. The association between *M*. *hyorhinis* and *M*. *hyosynoviae* detection was evaluated by Pearson’s Chi-squared test as a non-parametric 2-sample test for equality of proportions, with Yates continuity correction for small sample size. Throughout the study, dam and piglets were tested separately, with separate results for each population, as both the independent variables were of interest for hypothesis testing. In addition, the number of dams with dual *M*. *hyorhinis* and *M*. *hyosynoviae* detection, *M*. *hyorhinis* or *M*. *hyosynoviae* detection only, and no detection at each week of sampling were also evaluated descriptively. Proportions were calculated as the number of *M*. *hyorhinis* or *M*. *hyosynoviae* detections divided by the total number of pigs tested in the group in each week of sampling.

A comparison of two population proportions was also performed in order to evaluate dam parity association with detection of *M*. *hyorhinis* and *M*. *hyosynoviae* in tonsillar swabs at each week of sampling. Moreover, the relative risk of positive *M*. *hyorhinis* detection at week three post-farrowing was determined considering previous positive *M*. *hyorhinis* detection at week one post-farrowing as exposure. A similar analysis was also performed for *M*. *hyosynoviae* detection. Furthermore, proportions of positive detection in piglet tonsillar swabs prior to weaning were compared with positive results of individual lameness scores (score of 1 or greater) on each week of sampling after weaning by Chi-square test. Selected piglets were individually scored for lameness (0–4) and the weekly mean scores were individual based, instead of pen based. Statistical significance was considered when *p*-values were lower than 0.05. Statistical analysis and graphical representation were performed in R v3.2 (R Core Team) [24].

## Results

**Fig 2:**
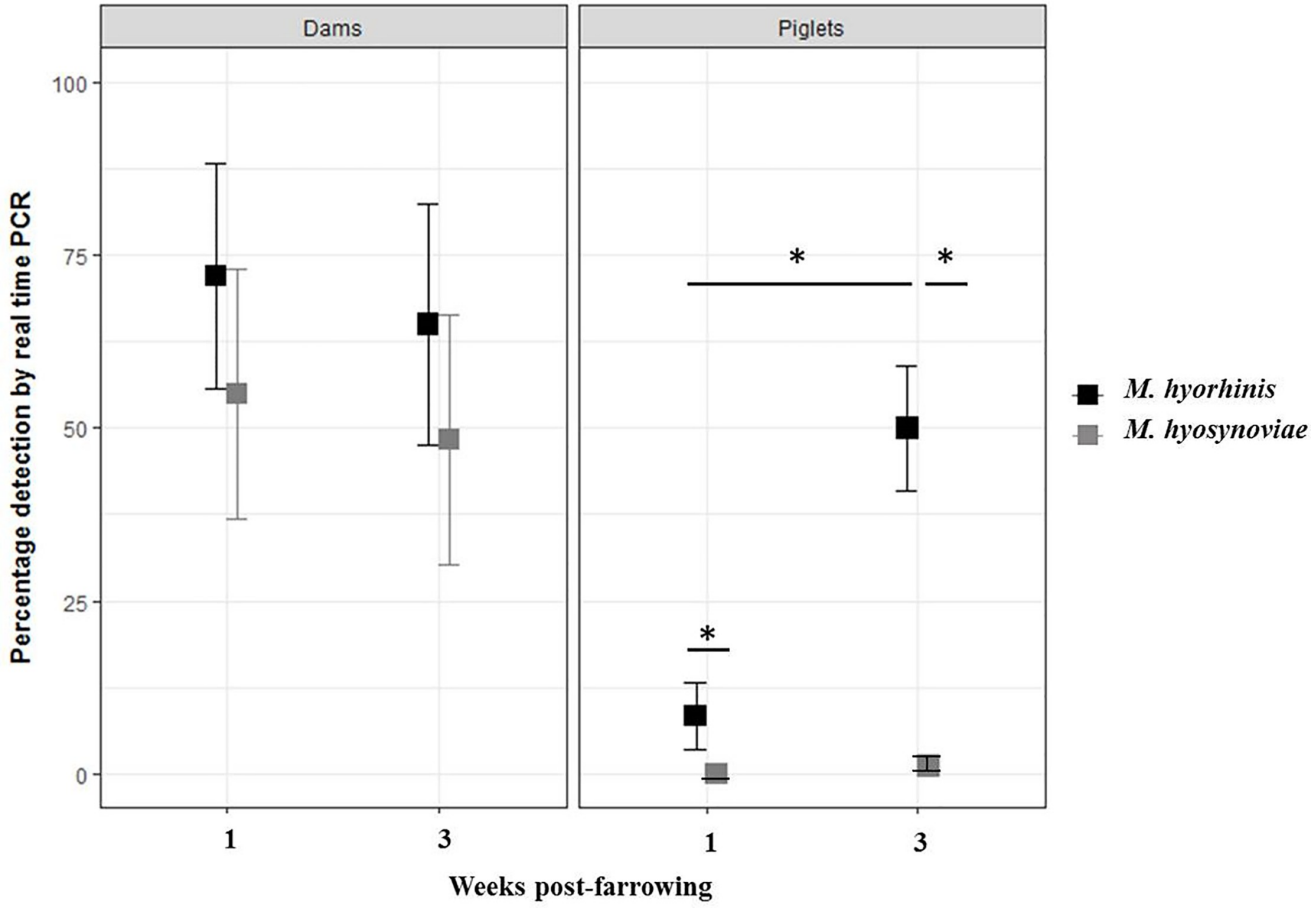
*Mycoplasma hyorhinis* and *Mycoplasma hyosynoviae* detection (%) in tonsillar swabs collected from dams and piglets tested by PCR at weeks one and 3 post-farrowing. Proportion of dams and piglets detected positive with *M*. *hyorhinis* are represented using black squares and dams and piglets positive for *M*. *hyosynoviae* are represented with grey squares. Error bars depict the 95% confidence interval. Detection of *M*. *hyorhinis* was significantly higher than *M*. *hyosynoviae* detection in tonsillar swabs collected from piglets at weeks one (*M*. *hyorhinis*: 8.3%; 95% CI: 3.4–13.2% % and *M*. *hyosynoviae*: 0%; 95% CI: 0–0.034) and three (*M*. *hyorhinis*: 50%; 95% CI: 41–58.9% and *M*. *hyosynoviae*: 0.9%; 95% CI: 0–2.6%) of sampling (*p*<0.05). *M*. *hyorhinis* detection in piglets was significantly higher at week 3 (50%; 95% CI: 41–58.9%) compared to week 1 (8.3%; 95% CI: 3.4–13.2%) (*p*<0.05). * *p*<0.05 between pathogens or ages at collection.

The detection pattern of *M*. *hyorhinis* and *M*. *hyosynoviae* in tonsillar swabs collected from dams and piglets tested by real-time PCR is shown in Fig 2. In week one post-farrowing, *M*. *hyorhinis* was detected in tonsillar swabs of 72% (95% CI: 55.7–88.3%) of dams and 8.3% (95% CI: 3.4–13.2%) of piglets. In the case of *M*. *hyosynoviae*, 55% (95% CI: 36.9–73.1%) of tonsillar swabs from dams were detected positive, one-week post-farrowing, while no piglets were detected positive the same week. At week three post-farrowing, *M*. *hyorhinis* was detected in tonsillar swabs of 65% (95% CI: 47.6–82.4%) of dams and 50% of piglets (95% CI: 41–58.9%) whereas *M*. *hyosynoviae* was detected in tonsillar swabs of 48.3% (95% CI: 30.1–66.5%) of dams and 0.9% (95% CI: 0–2.6%) of piglets.

The detection of *M*. *hyorhinis* was significantly higher in tonsillar swabs of piglets at week three compared to week one (*p*<0.05). Detection of *M*. *hyorhinis* was significantly higher than *M*. *hyosynoviae* detection in piglet tonsillar swabs at both weeks of sampling (*p*<0.05). The proportion of dams detected with *M*. *hyorhinis* or *M*. *hyosynoviae* at either week of sampling was not statistically significant (*p*>0.05).

**Fig 3:**
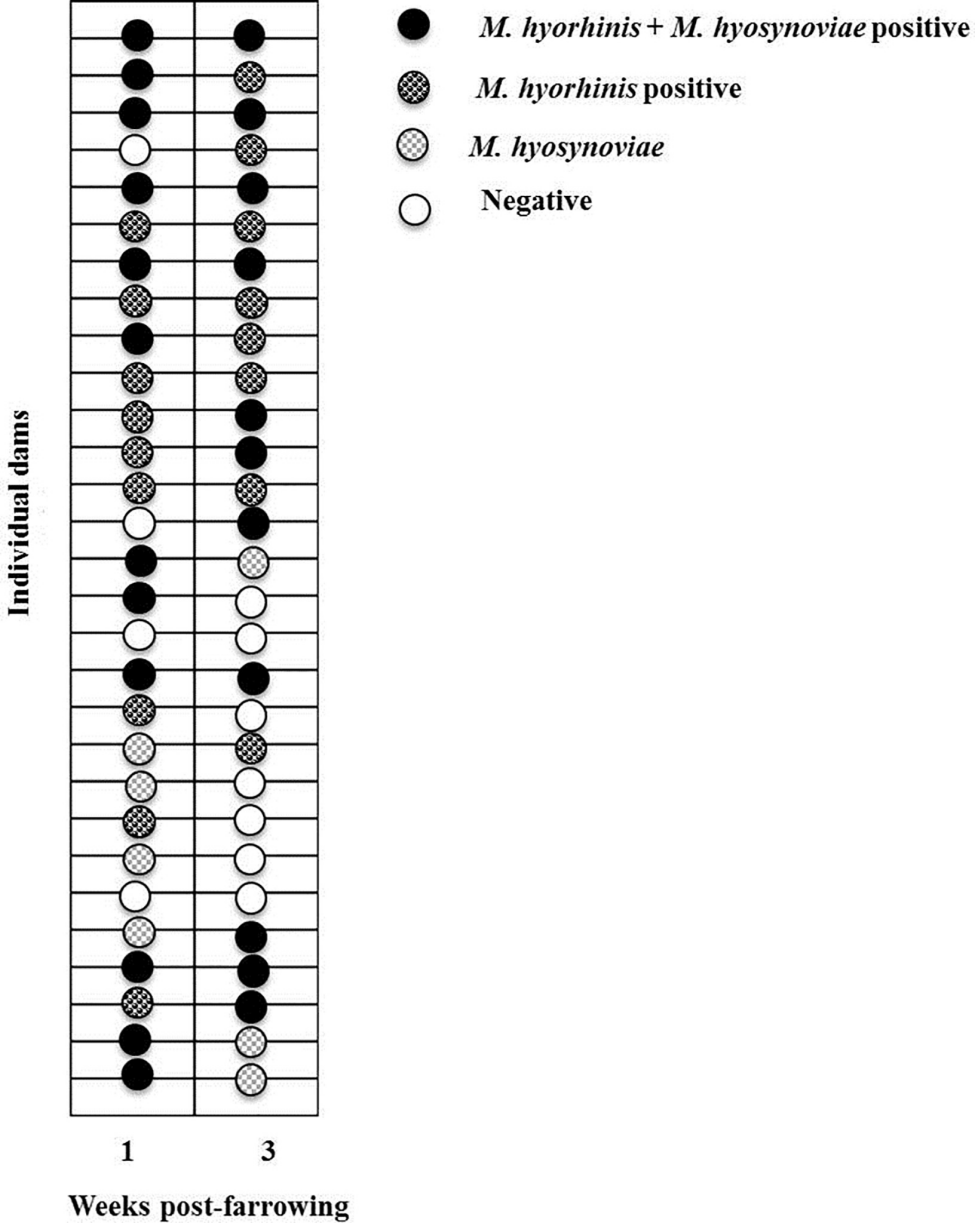
Individual *M*. *hyorhinis* and *M*. *hyosynoviae* detection in tonsillar swabs collected from 29 dams at weeks one and three post-farrowing. Each circle denotes individual dam and fill pattern indicates the detection pattern.

Individual *M*. *hyorhinis* and *M*. *hyosynoviae* detection in tonsillar swabs collected from dams at each week post-farrowing are shown in Fig 3. Among 29 dams, in both weeks of sampling, 51.7% (15/29) remained positive for *M*. *hyorhinis* in tonsillar swabs, whereas 34.5% (10/29) were detected positive for *M*. *hyosynoviae*. Twenty-one percent dams (6/29) were detected *M*. *hyorhinis* positive only at week one, 13.8% (4/29) were positive at week three alone, and 13.8% (4/29) remained negative for *M*. *hyorhinis* detection throughout the study. Similarly, in the case of *M*. *hyosynoviae*, 20.6% (6/29) of dams were positive only at week one, 13.8% (4/29) positive at week three alone, and 31% (9/29) of dams were negative at both weeks of sampling. In week one, 41.3% (12/29) of dams were detected positive for both *M*. *hyorhinis* and *M*. *hyosynoviae*, whereas in week three only 37.9% (11/29) were dual detected positive.

Considering *M*. *hyorhinis* detection results at both weeks in tonsillar swabs collected from dams, the estimated relative risk of positive detection in week three post-farrowing was 1.4286 (95% CI: 0.6789- 3.0059) if dams were positive at week one (*p<*0.05). In other words, dams with positive detection at week one were 1.43 times at risk of a positive detection at week three compared to dams with no detection at week one. In the case of *M*. *hyosynoviae* detection, the estimated relative risk of positive detection in week three post farrowing was 1.78 (95% CI: 0.88–3.6), if positive at week one although these differences were not statistically significant.

**Fig 4:**
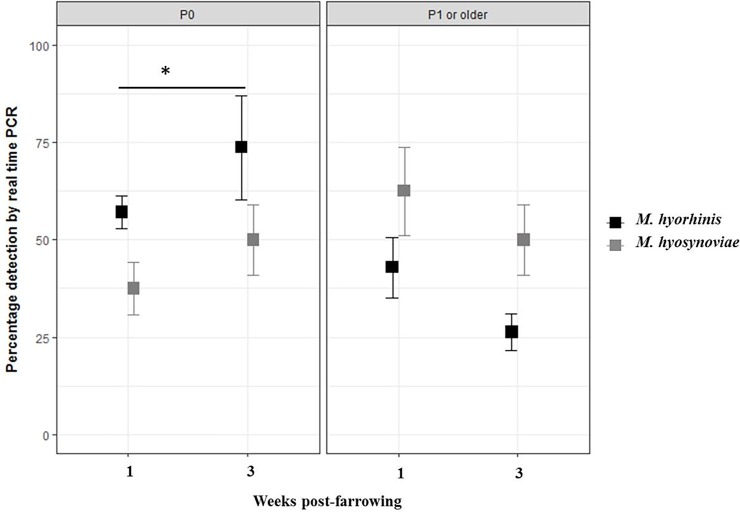
Percentage of first (P0) and multiple parity dams (P1 or older) with detection of genetic material for *Mycoplasma hyorhinis* and *M*. *hyosynoviae* in tonsillar swabs by real-time PCR at weeks one and three post-farrowing. Proportion of dams detected positive with *M*. *hyorhinis* are represented using black squares and dams positive for *M*. *hyosynoviae* are represented with grey squares. Error bars depict the 95% confidence interval limit. *Detection of *M*. *hyorhinis* in first parity dams was significantly higher at week three post-farrowing compared to week one (*p*<0.05).

The detection pattern of *M*. *hyorhinis* and *M*. *hyosynoviae* in tonsillar swabs collected from dams with respect to parity is shown in Fig 4. Among the 29 dams sampled, 14 were first-parity dams (P0) and 15 were multiple parity dams (P1 or older). In week one post-farrowing, among the total *M*. *hyorhinis* positive dams (n = 21), first parity dams (P0) corresponded to 57.1% (12/21) and multiple parity dams (P1 or older) corresponded to 42.9% (9/21) of *M*. *hyorhinis* detection in tonsillar swabs. Among the total *M*. *hyosynoviae* positive dams (n = 16), 37.5% (6/16) was detected from the first parity dams whereas multiple parity dams corresponded to 62.5% (10/16) of *M*. *hyosynoviae* detection in tonsillar swabs.

In week three post-farrowing, first parity dams contributed to 73.7% (14/19) of *M*. *hyorhinis* detection in tonsillar swabs whereas multiple parity dams contributed 26.3% (5/19) of the total *M*. *hyorhinis* positive dams (n = 19). Similarly, first parity dams corresponded to 50% (7/14) of *M*. *hyosynoviae* detection in tonsillar swabs and multiple parity dams corresponded to 50% (7/14) of the total *M*. *hyosynoviae* positive dams (n = 14). *M*. *hyorhinis* detection was significantly higher in tonsillar swabs collected from first parity dams when compared to multiple parity dams (*p*<0.05). Nevertheless, no association was observed between *M*. *hyorhinis* and/or *M*. *hyosynoviae* detection and parity at either week of sampling.

Mean individual lameness scores measured in the wean-to-finish phase are shown in Table 1. The estimated relative risk of developing lameness at least once during post-weaning (from week 5 to week 22) was 1.862 (95% CI: 1.508–2.463) if the piglets were detected positive for *M*. *hyorhinis* at week three. There was a significant association between positive detection of *M*. *hyorhinis* by real-time PCR in tonsillar swabs at week three and a positive lameness score during its post-weaning age (r = 0.44; *p*<0.05). However, the association between positive detection of *M*. *hyosynoviae* and lameness score in post-weaning was not established due to fewer numbers of positive cases in week three. Therefore, from the estimated relative risk of lameness reported in piglets in the *M*. *hyorhinis* positive group, the proportion of events are statistically different after controlling for the week of detection (*p*<0.05) compared to that of the *M*. *hyosynoviae* positive group.

**Table 1:** Mean individual lameness scores in piglets at different weeks of age.

Week of age	Mean lameness score	Percentage of piglets lame (lameness score ≥1)
5	0.228	20.4
7	0.214	20.4
10	0.089	5.6
13	0.075	5.6
16	0.056	5.6
19	0.075	7.4
22	0.134	11.1

Means do not exceed 0.5 on a 0–4 scale due to the small proportion of piglets with a score of 1 or greater. Individual lameness scores were obtained by evaluating lameness in each selected pig. Lameness score scale 0 to 4 (Nielsen et al., 2001).

## Discussion

This investigation was aimed to evaluate the detection of *M*. *hyorhinis* and *M*. *hyosynoviae* in dams and piglets sampled in farrowing rooms using tonsillar swabs real-time PCR testing. Dams appeared to be consistently positive for both *M*. *hyorhinis* and *M*. *hyosynoviae*. On the other hand, *M*. *hyorhinis* and *M*. *hyosynoviae* were detected in a small proportion of piglets in week one, except for *M*. *hyorhinis* immediately prior to weaning, which was detected in half of the sampled piglet population.

*M*. *hyorhinis* was detected in a higher proportion of first parity dams than in multiple parity dams in both weeks of sampling, although this difference was only significant on week three of sampling. Detection of *M*. *hyosynoviae*, however, was higher in multiple parity dams in the first week of sampling, yet an increase in PCR detection was observed in first parity dams in week three. The pattern of increasing detection between weeks one and three post-farrowing observed for both microorganisms in first parity dams may reflect a more recent transmission event and consequent colonization. Nevertheless, the role of first parity dams in the *M*. *hyorhinis* and *M*. *hyosynoviae* detection dynamics has not been previously reported. Furthermore, dams were detected with both microorganisms simultaneously at a high proportion in weeks one (41.3%) and three (37.9%) post-farrowing. Yet the association between *M*. *hyorhinis* and *M*. *hyosynoviae*, and the possible competitive mechanisms of dual colonization has to be further elucidated. To our knowledge, this is one of the first investigations to evaluate *M*. *hyorhinis* and *M*. *hyosynoviae* dual detection in dams and their offspring.

*M*. *hyorhinis* was detected in piglets at both weeks of sampling prior to weaning, although in a significantly higher proportion at week three post-farrowing. In contrast, *M*. *hyosynoviae* was only detected in piglets at week three post-farrowing, and in a very low proportion. Hence, these results could support the hypothesis that the colonization status of piglets in lactation might influence the timing of disease development, which has been reported in the nursery stage for *M*. *hyorhinis* [9,25], and in the finishing stage for *M*. *hyosynoviae* [2,7,8]. This was in line with the observed high association of *M*. *hyorhinis* detection and lameness after weaning in the study. However, the actual cause of the lameness was not determined.

It is important to take into account that maternally derived antibodies may play a protective role against *M*. *hyosynoviae* colonization prior to weaning [13,26], delaying the transmission between piglets post-weaning [14,26], with few colonized piglets acting as carriers. It has been shown that *M*. *hyorhinis* maternally derived antibodies decrease at approximately seven weeks of age, whereas antibodies agains *M*. *hyosynoviae* seem to persist until 11 weeks of age [5], which may contribute to the earlier colonization by *M*. *hyorhinis*. Nonetheless, detection of *M*. *hyorhinis* and *M*. *hyosynoviae* specific and maternally-derived antibodies was not performed in this study.

In commercial settings, various sample types have been employed for detection of *M*. *hyorhinis* and *M*. *hyosynoviae* using real-time PCR. Neto et al. [3] suggested that pen-based oral fluids and tonsillar scrapings collected from post-weaning pigs were capable of detecting both pathogens; while, nasal swabs were identified as a sensitive sample for *M*. *hyorhinis* detection only. Colonization with *M*. *hyorhinis* in dams and piglets prior to weaning has been reported to be lower than 10% in nasal swabs tested by real-time PCR [16, 23]. However, our results revealed a higher proportion of *M*. *hyorhinis* detection using the same PCR technique, on tonsillar swabs. Interestingly, when evaluated for another swine mycoplasma (*M*. *hyopneumoniae)*, nasal swabs were reported as a more sensitive sample when compared with tonsillar swabs [27]. Nevertheless, it is important to note that *M*. *hyopneumoniae* is not considered a commensal microorganism of the nasal cavities and is mainly detected in the lower respiratory tract of pigs.

Despite the diagnostic sensitivity of tonsillar swabs for detection of *M*. *hyorhinis* and *M*. *hyosynoviae* not being yet reported, a potential hypothesis is that the sensitivity would fall between that of nasal swabs and tonsillar scrapings, with the latter likely identified as the most sensitive sample at the individual pig level [3]. Due to the collection technique, tonsillar swabs may be considered a less invasive *in vivo* sample than tonsil scrapings, yet, less tonsil tissue may be harvested in the process of tonsillar swabs collection. In addition, the association between tonsillar detection and disease development needs further investigation, considering that the tonsils are a selective tissue for *M*. *hyorhinis and M*. *hyosynoviae* natural colonization and may not imply disease association. Variation from the observations in this study is also expected in different farms and systems, as sample collection can carry intrinsic result diversity, although detection of *M*. *hyorhinis* or *M*. *hyosynoviae* is extremely common in most swine farms. Moreover, it is important to note that this study was performed in a herd with previous history of lameness.

Control of *M*. *hyorhinis* and *M*. *hyosynoviae* in the field can be challenging due to the commensal nature of these microorganisms [20], and their potential multi-factorial aspect of disease presentation [2,19]. *Mycoplasma hyorhinis* and *M*. *hyosynoviae* transmission would likely continue among piglets in later production stages. Information on *M*. *hyorhinis* and *M*. *hyosynoviae* epidemiology are sparse and efforts must be made to improve the understanding of the colonization dynamics and disease processes of these microorganisms in order to aid decision-making by practitioners and swine producers in the application of control strategies.

## Conclusions

This investigation aimed to assess for the first time *M*. *hyorhinis* and *M*. *hyosynoviae* dual detection in dams and their offspring prior to weaning. Under the conditions of this investigation, dams appeared to be consistently positive for both *M*. *hyorhinis* and *M*. *hyosynoviae* prior to weaning. In contrast, higher detection was observed in piglets at week three, in comparison to week one post-farrowing, with *M*. *hyorhinis*, while detection of *M*. *hyosynoviae* was remarkably minimal. The relative risk of developing lameness in postweaning piglets was highly associated with the detection of *M*. *hyorhinis* at three weeks of age. In addition, a high detection of *M*. *hyorhinis* in dams and piglets was obtained in this study with the use of tonsillar swabs as a sample type.

## Supporting information

S1 Table Roos et al—PCR data—All treatments.xlsx. The supporting information provides raw CT values from *M*. *hyorhinis* and *M*. *hyosynoviae* PCR detection, as well as an overall summary of the data collected.
(XLSX)
